# Exploiting the relevance of CA 19-9 in pancreatic cancer

**DOI:** 10.20517/2394-4722.2020.70

**Published:** 2020-09-17

**Authors:** Syaza Salleh, Anita Thyagarajan, Ravi P. Sahu

**Affiliations:** Department of Pharmacology and Toxicology, Boonshoft School of Medicine Wright State University, Dayton, OH 45435, USA

**Keywords:** Pancreatic ductal adenocarcinoma, tumour biomarker, carbohydrate antigen 19-9, treatment efficacy

## Abstract

Pancreatic ductal adenocarcinoma (PDAC) is currently the fourth most common cause of cancer-related deaths in the United States. It has a poor prognosis and remains a difficulty to treat malignancy. Over the past several decades, significant efforts have been directed towards developing new approaches to enhance the efficacy of therapeutic regimens for PDAC treatment. In recent years, the measurement of serum carbohydrate antigen 19-9 (CA 19-9) has become one of the most validated and extensively used tumour biomarkers for PDAC. In particular, serum CA 19-9 levels have been explored as a validated tool to predict either the signs of disease progression or the response to treatment. However, despite its clinical relevance, the implications on diagnosis or accurately predicting tumour resectability, and monitoring disease symptoms in PDAC patients remains limited. This current review highlights the recent updates on the applicability of CA 19-9, its exploitation, and challenges in predicting the treatment efficacy and responses in PDAC patients.

## INTRODUCTION

Pancreatic ductal adenocarcinoma (PDAC) currently represents the fourth leading cause of cancer-related mortality, and is associated with a poor prognosis and a median survival of 6–9 months^[1–3]^. Research into an effective treatment option for pancreatic cancer has long eluded the global research community. The majority of patients are usually diagnosed at advanced or metastatic stages where tumour cells have migrated from their local environment to distant sites^[4,5]^. Importantly, this cancer type remains a hardto-treat malignancy mostly due to the development of tumour resistance mechanisms to the conventional therapeutic approaches, which have been correlated with overall decreased survival rates^[4,5]^. Despite these ongoing challenges, in recent years, incremental improvements were observed in the overall survival rate due to the emergence of combination chemotherapy regimens^[6,7]^. For example, gemcitabine plus nab-paclitaxel, and FOLFIRINOX [a combination of 5-fluorouracil (5-FU), leucovorin, irinotecan, and oxaliplatin] show clinically meaningful improvements compared with gemcitabine monotherapy for PDAC patients^[6,7]^.

In the last decade, different biomarkers have been explored to identify a simpler tool for pancreatic cancer diagnosis and prognosis^[8,9]^. The development of tumour biomarkers are essential to precisely detect patients at their early stage of tumour growth, as well as to evaluate the treatment responses so that the current therapeutic strategies can be further improved^[8–11]^. Among various validated tools, alcohol dehydrogenase (ADH) isoenzymes, aldehyde dehydrogenase (ALDH) enzyme as well as carbohydrate antigen 19-9 (CA 19-9) have been explored as the most common tumour markers for pancreatic cancer^[8–14]^. ADH and ALDH enzymes catalyse alcohol metabolism via the oxidative pathway, which eventually results in the generation of carcinogenic acetaldehyde. Given the importance of these biomarkers, studies by Jelski *et al.*^[12]^ compared the activities of ADH isozymes and ALDH in pancreatic cancer *vs*. normal tissues, as well as their differences between drinkers and non-drinkers. The authors observed that the class III ADH isoenzyme activity was significantly increased in pancreatic cancer tissue (14.03 mU/L) compared to the healthy tissue (11.45 mU/L). However, no significant differences were noticed in the activities of other ADH isoenzymes and ALDH between pancreatic cancer and normal cells^[12]^. Overall, these findings indicated that ADH and ALDH mediated oxidative pathways of ethanol metabolism are not critical players in pancreatic carcinogenesis^[12]^. However, considering the fact of increased ADH class III enzyme activity in pancreatic cancer, the same group analysed ADH isozymes and ALDH activities in the serum samples of pancreatic cancer patients^[13]^. The authors observed a significant increase in ADH class III isoenzyme activity (13.52 mU/L) in pancreatic cancer patients compared to the control group (11.08 mU/L)^[13]^. While total ADH activity was also significantly higher in cancer patients, no changes were noticed in other ADH isozymes as well as ALDH activities^[13]^. Notably, a similar analysis in a large cohort of pancreatic cancer patients also demonstrated significantly increased activity of serum ADH class III isozyme (14.03 mU/L) compared to healthy controls (11.45 mU/L), respectively^[14]^. Moreover, total serum ADH activity was also found to be increased compared to healthy controls^[14]^. Overall, these studies suggested a potential role for ADH, particularly the class III isoenzyme as a biomarker of pancreatic cancer.

In recent years, CA 19-9, also known as Sialylated Lewis antigen (Sla) has emerged as one of the most-studied biomarkers for PDAC, which was characterized by a monoclonal antibody produced by hybridoma technology^[8–11]^. As some mucins could contribute to cancer progression via mechanisms including the induction of T cell apoptosis, which later affects the anti-cancer immune response^[10,11]^, the human colorectal carcinoma cell line SW1116 was immunized with spleen cells of a mouse as a mucin-like product^[11]^. The authors of this study characterized that CA 19-9 works by detecting an antigen of a carbohydrate on numerous protein carriers. Proteins that carry CA 19-9 were then identified by immunoprecipitating CA 19-9 from the pooled sera. In addition, mass spectrometry was also used to identify CA 19-9 associated proteins. Notably, various assays such as antibody arrays, western blot, kininogen, and apolipoprotein E by antibody arrays confirmed the presence of CA 19-9 antigen on apolipoprotein B-100^[10,11]^.

### Mechanism of CA 19-9 biogenesis

CA 19-9 is generated by deregulated glycosylation, a process that enzymatically links glycan sugars to cellular lipids and proteins^[15–17]^. In the normal pancreas, glycosylated proteins play various crucial functions including protecting and lubricating the pancreatic ducts^[15–17]^. However, during cancer progression aberrant glycosylation, one of the hallmarks of cancer, occurs due to numerous modifications that result in the formation of various glycosylated residues such as CA 19-9/(SIa) which has emerged as a potential biomarker^[15–17]^.

To emphasize the clinical relevance of this molecule, the survival rates of PDAC patients have been shown to be inversely related to the levels of CA 19-9^[18,19]^. In these reports, increased levels of CA 19-9 were found to be associated with unresectable lesions or more numbers of advanced tumours, which indicates a poor prognosis for PDAC patients^[18,19]^. Given the importance of this biomarker, studies conducted by Isacoff *et al*.^[20]^ have demonstrated that patients with normal levels of CA 19-9 (i.e., 36 U/mL or less) during the initial course of therapy had higher median survival rate compared to those who had elevated CA 19-9 levels (more than 36 U/mL). Furthermore, the median survival of PDAC patients on therapy who had a reduction in CA 19-9 levels by 90% was significantly longer than those patients with a less than 90% reduction in the level of this biomarker^[20]^. However, since CA 19-9 is a tumour-associated but not a tumour-specific antigen, its implications are not pathognomonic for pancreatic cancer. It is known to be synthesized by the gall bladder, epithelial cells of the pancreas, stomach, and biliary ducts, which explains the increased level of CA 19-9 in the benign as well as advanced conditions of many malignancies^[21–24]^. In this current review, we highlight the recent updates, and therapeutic potential, of CA 19-9, with an emphasis on experimental and clinical studies, as well as its importance as a prognostic biomarker in pancreatic cancer.

## IMPLICATION OF CA 19-9 IN PDAC

### *In vivo* studies

Several studies have highlighted the role of CA 19-9 in PDAC models^[25,26]^. Wagner *et al*.^[27]^ conducted an *in vivo* study using three orthotopic xenograft mouse models to examine the efficacy of targeted immunocytokine L19-IL-2 in pancreatic cancer with the goal of assessing CA 19-9 as a biomarker for tumour progression when treated with L19-IL-2. Treatment with L19-IL-2 resulted in a significant reduction in the serum levels of CA 19-9, but no changes were observed with either IL-2 or L19 alone treatments. In contrast, there was a significant increase in the serum levels of CA 19-9 in the untreated control group of mice. This indicates a significant correlation between the serum levels of CA 19-9 and tumour volume of PDAC, and that a positive response of L19-IL-2 on CA 19-9 was observed against PDAC^[27]^. Overall, these results corroborate the crucial role of CA 19-9 as a biomarker to assess the treatment responses for PDAC [Table 1].

### Human studies

#### Applicability of CA 19-9 levels to determine therapeutic and prognostic responses in PDAC

There have been numerous studies investigating the clinical relevance of CA 19-9 in pancreatic cancer, and to define its applicability in predicting either the responses or resistance to various treatments^[28–30]^. A summary of these investigations is given in Table 2. Kieler *et al*.^[28]^ evaluated CA 19-9 levels in response to chemotherapy with nanoliposome irinotecan (nal-IRI) plus 5-fluorouracil/leucovorin (5-FU/LV) as a second-line treatment for PDAC and compared it with oxaliplatin plus fluoropyrimidines. The findings revealed that at the start of therapy, the median progression-free survival (PFS) was significantly longer in patients who received nal-IRI plus 5 FU/LV compared to the median PFS in patients treated with oxaliplatin plus fluoropyrimidines. In addition, the median overall survival (OS) of patients with CA 19-9 levels over 772.8 kU/L receiving nal-IRI plus 5 FU/LV was significantly higher compared to those who were treated with oxaliplatin plus fluoropyrimidines. This study indicated the effectiveness of nal-IRI plus 5-FU/LV treatment in PDAC when compared with oxaliplatin plus fluoropyrimidines, and that increased CA 19-9 response was associated with better therapeutic outcomes.

In another report, Li *et al*.^[29]^ performed a prospective study to determine the therapeutic response to modified-FOLFIRINOX and correlated it with CA19-9 levels in patients with metastatic PDAC. The study demonstrated that patients receiving modified-FOLFIRINOX therapy had decreased levels of CA 19-9 and that this decrease in CA 19-9 was found to be associated with a significantly longer median OS of patients^[29]^. In another study, Aoki *et al*.^[30]^ determined the prognostic significance of decreased CA 19-9 after receiving neoadjuvant therapy in patients with PDAC. The authors found no increase in CA 19-9 levels in patients receiving neoadjuvant therapy and that the responder group demonstrated a lower risk of hepatic recurrence compared to the non-responder group^[30]^. Overall, these studies indicate the importance of evaluating CA 19-9 levels as a prognostic biomarker in monitoring the response to treatments for PDAC.

Chiorean *et al*.^[31]^ conducted a randomized phase III trial (MPACT) to evaluate the effect of weekly *nab*-paclitaxel plus gemcitabine over gemcitabine alone on CA 19-9 levels at eight-week intervals as a predictor of OS in metastatic pancreatic cancer patients. The authors observed that patients treated with nab-paclitaxel plus gemcitabine had better outcomes compared with gemcitabine alone and that the improved outcomes were associated with decreased levels of CA 19-9 compared to increased or static levels. In the same context, there was a statistically significant survival advantage for patients receiving nab-paclitaxel plus gemcitabine *vs*. gemcitabine alone^[31]^. In addition, an abrupt decrease in CA 19-9 levels was observed in each of the two treatment arms during the initial eight weeks period of treatment^[31]^. Moreover, prolonged OS and PFS has also been found to be associated with a decline in CA 19-9 levels^[31]^. Overall, these findings confirmed the applicability of CA 19-9 as an early biomarker to access the anti-tumour efficacy of therapeutic agents for metastatic pancreatic cancer patients.

### Advantages of serum CA 19-9 levels as a biomarker for defining the surgical resectability in PDAC patients

Given that the CA 19-9 levels were positively correlated with the OS, several studies evaluated an association between CA 19-9 and tumour resectability. While surgical resection remains one of the potential curative options for PDAC, many cases are not amenable to surgical resection at the time of evaluation^[32–35]^. For that reason, identifying unresectable patients preoperatively is vital to avoid undesired surgery^[32–35]^. For this reason, the use of serum CA 19-9 levels pre-operatively has been extensively evaluated to determine resectability in pancreatic cancer patients^[32,33,36–40]^.

Heger *et al*.^[40]^ investigated the dynamics of CA 19-9 levels during neoadjuvant treatment (NT) in order to predict resectability and survival. The authors noticed a significant correlation between the reduced levels of CA 19-9 with resectability and OS. Importantly, a significant difference in CA 19-9 levels was observed following the treatment with FOLFIRINOX (FOL) as patients treated with this regimen had a significantly higher resection rate compared to those treated by gemcitabine-based NT^[40]^. Furthermore, the combination of NT and FOL was found to be associated with a significantly improved survival rate among resected pancreatic cancer patients^[40]^. This study corroborates findings by Bolton *et al*.^[41]^ who delineated their experience and correlated the findings between borderline resectable pancreatic cancer (BRPC) *vs*. initially resectable pancreatic cancer (IRPC) patients receiving neoadjuvant therapy. While no significant differences were observed in the OS responses between IRPC and BRPC prior to multiagent neoadjuvant therapy, BRPC patients treated with neoadjuvant therapy experienced increased OS^[41]^. Also, patients with a high College of American Pathologists (CAP) score or a less than 50% increase in CA19-9 levels had longer survival than patients with a low CAP score and > 50% CA19-9 levels^[41]^. Importantly, compared to all other treatments, FOLFIRINOX treatment followed by neoadjuvant therapy significantly improved the survival response in BRPC patients^[41]^. Overall, these findings indicate that multiagent neoadjuvant therapy is associated with an increased survival rate, which is correlated with a decrease in CA 19-9 levels when compared to other neoadjuvant therapies.

Notably, these findings appear to be consistent with the studies by van Veldhuisen *et al*.^[42]^, who evaluated the diagnostic accuracy of serum CA 19-9 in combination with RECIST-response on CT imaging to predict the resectability of locally advanced pancreatic cancer (LAPC) following induction chemotherapy. The authors reported that all patients with RECIST-regressive disease exhibited a significant decrease in the serum levels of CA 19-9 following induction chemotherapy, which indicated a positive correlation between CA 19-9 levels and RECIST-regressive disease. Importantly, an elevation in the CA 19-9 level was also found to be significantly associated with decreased survival^[42]^.

Another study conducted by Xu *et al*.^[43]^ evaluated the efficacy of adjuvant chemoradiotherapy by assessing the postoperative serum levels of CA 19-9. In this study, adjuvant chemoradiotherapy was found to be associated with improved surgical outcomes in patients with increased levels of this biomarker, but not in patients with the normal levels of CA 19-9^[43]^. In addition, a significant improvement in surgical outcomes was noticed in patients with increased levels of serum CA 19-9 with negative lymph nodes^[43]^. Overall, these findings suggest the importance of evaluating preoperative CA 19-9 levels for monitoring the therapeutic responses of various regimens, as well as determining the resectability of the disease.

#### Benefits of serum CA 19-9 levels as a biomarker for determining the surgical resectability in PDAC patients with jaundice

Santucci *et al*.^[34]^ performed a study to determine if serum CA 19-9 levels can be exploited as a biomarker for predicting the resectability of PDAC, mainly in jaundiced patients. The authors reported that the mean CA 19-9 level in patients with the resectable disease was significantly lower compared to those with locally-advanced or metastatic disease^[34]^. It has also been observed that the ability of CA 19-9 in precisely predicting resectability was 0.886 when evaluated with the area under the Receiver Operating Characteristic (ROC) curve. This implies that the resectability in 88.6% of patients was accurately predicted by the serum CA 19-9 level. Concurrently, the area under the ROC curve in patients with jaundice corresponded well with data obtained for non-jaundiced patients^[34]^. These findings suggested that CA 19-9 can be explored as an accurate predictive biomarker to assess the resectability of PDAC patients with jaundice.

In a separate study, Choi *et al*.^[44]^ developed an integrated predictive model to determine the long-term survival in LAPC patients treated with chemoradiotherapy (CRT). The authors reported that pre-CRT CA 19-9, post-CRT CA 19-9, and a decline in CA 19-9 levels were among the significant factors that contributed to the favourable PFS^[44]^. It has been demonstrated that high-dose radiation, a decline in CA 19-9 levels, and surgical resection after receiving CRT, were all significantly correlated with longer OS^[44]^. Overall, these findings imply the crucial role of CA 19-9 as a biomarker for developing a nomogram to help determine the patients for CRT, and aiding clinical decision making.

## ONGOING CHALLENGES AND LIMITATIONS

There are a number of limitations that can confound the interpretation of CA 19-9 as a biomarker. Despite its remarkable contribution in clinical practice, the efficacy of CA 19-9 to be exploited as a biomarker remains controversial as it is not exclusively specific to this disease as numerous benign aetiologies can also deceptively increase the levels of CA 19-9^[43,45–51]^. In addition, this tumour biomarker has been shown to have a modest and low sensitivity (79%−81%) in symptomatic patients. This also limits its diagnostic utility, keeping CA 19-9 in the category of those clinical biomarkers whose implications need to be precisely considered as a screening tool keeping other limitations in place^[43,45–51]^.

## FUTURE PERSPECTIVES

Considering all the discussed limitations, several studies have suggested and evaluated the cut-off levels for serum CA 19-9 from 37 to 90 U/mL, which resulted in a potentially increased specificity of this biomarker to 95%^[43,44–51]^. In other respects, Jahan *et al*.^[52]^ conducted a study to explore the individual and combined Trefoil factors (TFFs) alone and in combination with CA 19-9 as a promising panel for detecting pancreatic cancer. TFFs are defined as secretory products of cells that produce mucin. The authors reported that a combination of TFFs with CA19-9 appeared to be a potential diagnostic tool for distinguishing between early-stage pancreatic cancer, benign controls, and chronic pancreatitis. Notably, the sensitivity of CA 19-9 was improved by its combination with TFFs^[52]^. In addition, the combination of TFFs and CA 19-9 was associated with an increased overall efficacy of CA 19-9 to discriminate early pancreatic cancer from chronic pancreatitis, which indicated the exceptional capabilities of TFFs to differentiate this highly aggressive disease at the early stages of progression.

## CONCLUSION

Based on the findings of various studies, CA 19-9 has proven to be an essential tool in the diagnosis of pancreatic cancer, and most importantly in monitoring patient responses to various treatment modalities. The validation of CA 19-9 as exhibiting excellent parameters, such as higher sensitivity and specificity compared to other biomarkers, has confirmed its potential as a promising clinical tool in this field. However, despite its undeniable benefits in the predictive analysis of treatment efficacy and responses in PDAC patients, there are still a number of limitations that warrant further investigation in the future.

## Figures and Tables

**Table 1.** Summary of *in vivo* studies defining the relevance of CA 19-9 in pancreatic cancer and treatment efficacy

Cell lines	Study design	Treatment(s)	Biomarker	Finding(s)	Ref.

Orthotopic xenograft mouse models, DanG and MiaPaca	To determine the efficacy of targeted immunocytokine L19-IL-2 in PDAC model by evaluating the CA 19-9 levels for assessing tumour progression	Targeted immunocytokine L19-IL-2	CA 19-9	There was a significant correlation between serum CA 19-9 concentrations and tumour volume. CA 19-9 levels were significantly reduced following L19-IL-2 treatment	[27]

**Table 2.** Summary of human studies defining the relevance of CA 19-9 in pancreatic cancer and treatment efficacy

No.	Study design	Treatment(s)	Biomarker	Finding(s)	Ref.

1.	To assess serum CA 19-9 levels with the response rates of chemotherapeutic agents, nanoliposome irinotecan (nal-IRI) plus 5-fluorouracil/leucovorin (5-FU/LV) as a second-line treatment option, and compare the responses with oxaliplatin plus fluoropyrimidines	Nanoliposome Irinotecan (nal-IRI) plus 5-fluorouracil / leucovorin (5-FU/LV) Oxaliplatin plus fluoropyrimidines	CA 19-9	CA 19-9 levels were significantly lower in patients receiving nal-IRI plus 5 FU/LV than those receiving oxaliplatin plus. Also, the median PFS and OS in patients who received nal-IRI plus 5 FU/LV were significantly longer compared to those receiving oxaliplatin plus fluoropyrimidines	[28]
2.	To evaluate CA 19-9 levels to determine modified-FOLFIRINOX responses in metastatic PDAC patients	Modified-FOLFIRINOX (FOL)	CA19-9	CA 19-9 levels were found to be decreased following modified-FOLFIRINOX treatment	[29]
3.	To determine the prognostic significance of CA 19-9 levels following neoadjuvant therapy (NT) in PDAC patients	Neoadjuvant therapy	CA 19-9	CA 19-9 stats indicated no increase following NT	[30]
4.	To study the changes in CA 19-9 levels as a predictor of OS in a randomized phase III trial	Nab-paclitaxel plus gemcitabine vs. Gemcitabine-alone	CA 19-9	Decreased CA 19-9 levels in each of the two treatments (nab-paclitaxel plus gemcitabine *vs* gemcitabine alone). Improved efficacy of combination treatment was observed with a decrease in CA 19-9 levels and patients had better outcomes compared with gemcitabine alone	[31]
5.	To investigate CA 19-9 levels and their dynamics during neoadjuvant treatment (NT) in predicting resectability and survival	FOLFIRINOX (FOL) vs. Gemcitabine-based NT	CA 19-9	Decreased CA19-9 levels were observed following treatment with FOL, and patients had significantly higher resection rates	[40]
6.	To determine CA 19-9 levels and correlate the results between borderline resectable pancreatic cancer (BRPC) vs. initially resectable pancreatic cancer (IRPC) receiving neoadjuvant therapy	Neoadjuvant therapy, FOLFIRINOX	CA 19-9	Patients with < 50% increase in CA19-9 levels had longer survival than patients with > 50% increase in CA19-9 levels. FOL treatment followed by neoadjuvant therapy significantly improved the survival response in BRPC patients	[41]
7.	To study the diagnostic accuracy of serum CA 19-9 in combination with RECIST-response on CT-imaging in predicting the resectability of locally advanced pancreatic cancer (LAPC) following induction chemotherapy	Induction chemotherapy	CA 19-9	There was a significant decrease in CA 19-9 levels following chemotherapy. An increase in the CA 19-9 level was significantly correlated with decreased survival	[42]
8.	To evaluate the efficacy of adjuvant chemoradiotherapy by assessing postoperative serum levels of CA 19-9	Adjuvant chemoradiotherapy	CA 19-9	Adjuvant chemoradiotherapy increased the surgical outcome in patients with increased levels of the CA 19-9 but not in patients with normal levels of CA 19-9	[43]
9.	To assess CA 19-9 serum levels as a biomarker in predicting the resectability of PDAC mainly in jaundiced patients	Any treatment	CA 19-9	The resectability in the majority of patients was accurately predicted by the serum CA 19-9 level. Also, the area under the ROC curve in patients with jaundice corresponded well with the findings obtained for non-jaundiced patients	[34]
10.	To develop an integrated predictive model to determine longer survival in locally advanced pancreatic cancer (LAPC) patients treated with chemoradiotherapy (CRT)	Chemoradiotherapy	CA 19-9	CA 19-9 was one of the significant factors which contributed to favourable PFS. In addition, decreased CA 19-9 levels and surgical resection following CRT treatment were significantly associated with higher OS	[44]

PDAC: pancreatic ductal adenocarcinoma; CA 19-9: carbohydrate antigen 19-9; TFFs: trefoil factors; LAPC: locally advanced pancreatic cancer; PFS: progression-free survival; RECIST: response evaluation criteria in solid tumours; CRT: chemo radiation therapy

## Data Availability

Not applicable.
